# Prosthetic hip joint infection by Bacillus Calmette-Guerin therapy following intravesical instillation for bladder cancer identified using whole-genome sequencing: a case report

**DOI:** 10.1186/s12879-021-05831-3

**Published:** 2021-02-05

**Authors:** Michael Riste, Pretin Davda, E. Grace Smith, David H. Wyllie, Martin Dedicoat, Simantini Jog, Steven Laird, Gerald Langman, Neil Jenkins, Jonathan Stevenson, Matthew K. O’Shea

**Affiliations:** 1 Department of Infectious Diseases, Heartlands Hospital, University Hospitals Birmingham NHS Foundation Trust, Birmingham, UK; 2 National Mycobacterial Reference Service, Public Health England, Heartlands Hospital, Birmingham, UK; 3 Department of Microbiology, Queen Elizabeth Hospital, University Hospitals Birmingham NHS Foundation Trust, Birmingham, UK; 4 Department of Cellular Pathology, University Hospitals Birmingham NHS Foundation Trust, Birmingham, UK; 5 Bone Infection Service, The Royal Orthopaedic Hospital NHS Foundation Trust, Birmingham, UK; 6 Institute of Immunology and Immunotherapy, University of Birmingham, Birmingham, UK

**Keywords:** *Mycobacterium bovis*, Bacillus Calmette-Guerin, Intravesical BCG therapy, Prosthetic joint infection, Whole-genome sequencing, Case report

## Abstract

**Background:**

Joint replacement is an effective intervention and prosthetic joint infection (PJI) is one of the most serious complications of such surgery. Diagnosis of PJI is often complex and requires multiple modalities of investigation. We describe a rare cause of PJI which highlights these challenges and the role of whole-genome sequencing to achieve a rapid microbiological diagnosis to facilitate prompt and appropriate management.

**Case presentation:**

A 79-year-old man developed chronic hip pain associated with a soft-tissue mass, fluid collection and sinus adjacent to his eight-year-old hip prosthesis. His symptoms started after intravesical Bacillus Calmette-Guerin (BCG) therapy for bladder cancer. Synovasure™ and 16S polymerase chain reaction (PCR) tests were negative, but culture of the periarticular mass and genome sequencing diagnosed BCG infection. He underwent a two-stage joint revision and a prolonged duration of antibiotic therapy which was curative.

**Conclusions:**

BCG PJI after therapeutic exposure can have serious consequences, and awareness of this potential complication, identified from patient history, is essential. In addition, requesting appropriate testing is required, together with recognition that traditional diagnostics may be negative in non-pyogenic PJI. Advanced molecular techniques have a role to enhance the timely management of these infections.

## Background

Joint replacement is a highly effective intervention and with an aging population and advancements in joint arthroplasty, demand is expected to increase [1]. Prosthetic joint infection (PJI) is one of the most serious complications of joint replacement surgery and is associated with significant morbidity. PJI affects 1–2% of all primary total hip and knee arthroplasties over the lifetime of the prosthetic joint and diagnosis and management can be difficult, prolonged and costly [2]. Diagnosis often relies on the results of multiple investigations including radiological, biochemical, microbiological and molecular. In the following case we describe a rare cause of PJI which highlights some of these diagnostic challenges.

## Case presentation

A 79-year-old male presented to a tertiary orthopaedic hospital in the United Kingdom with worsening pain and difficulty weight bearing localised to his previously asymptomatic total hip replacement. He had undergone an uncomplicated metal on polyethylene total hip replacement in 2008. Eighteen months prior to presentation to our unit he had developed a fluctuant swelling over the right hip. The swelling intermittently discharged serous fluid via a sinus which resolved spontaneously 2 months prior to presentation, after which he reported persistent pain in the joint and significant weight loss but no fevers or other systemic symptoms.

His past medical history included osteoarthritis of the spine, ischaemic heart disease, two myocardial infarctions, benign prostatic hyperplasia, pulmonary embolism following a trans-urethral resection of the prostate and transitional cell carcinoma of the bladder. For the latter he received six intravesical BCG vaccine instillations (Tice strain, Alliance Pharmaceuticals) over a 2 month period. Within a month of receiving the final BCG instillation the swelling over his right hip developed.

**Fig. 1:**
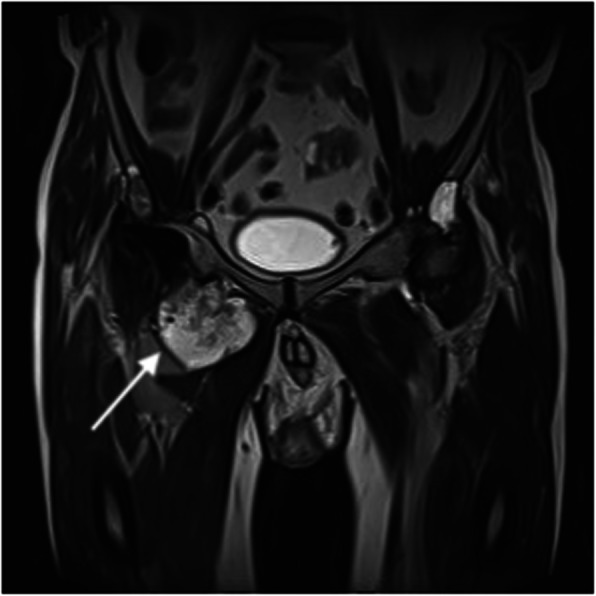
T1 MRI sequence of the pelvis showing the right total hip replacement with a large lobulated associated soft tissue mass arising in relation to the hip joint and the prosthesis (arrow), consistent with prosthesis-related pseudotumour

In view of the intermittently discharging sinus, the clinical diagnosis at initial orthopaedic assessment was PJI. Computed tomography of the pelvis showed a large collection around the right hip extending into the adductor compartment. The urinary tract appeared normal and there was no evidence that the collection was associated with any part of it. Magnetic resonance imaging (MRI) of the hip demonstrated a large, lobulated soft tissue mass arising in relation to the hip joint and prosthesis, which was felt to be consistent with a diagnosis of metallosis and pseudotumour due to aseptic lymphocyte-dominated vasculitis-associated lesions (Fig. 1) [3].

**Fig. 2:**
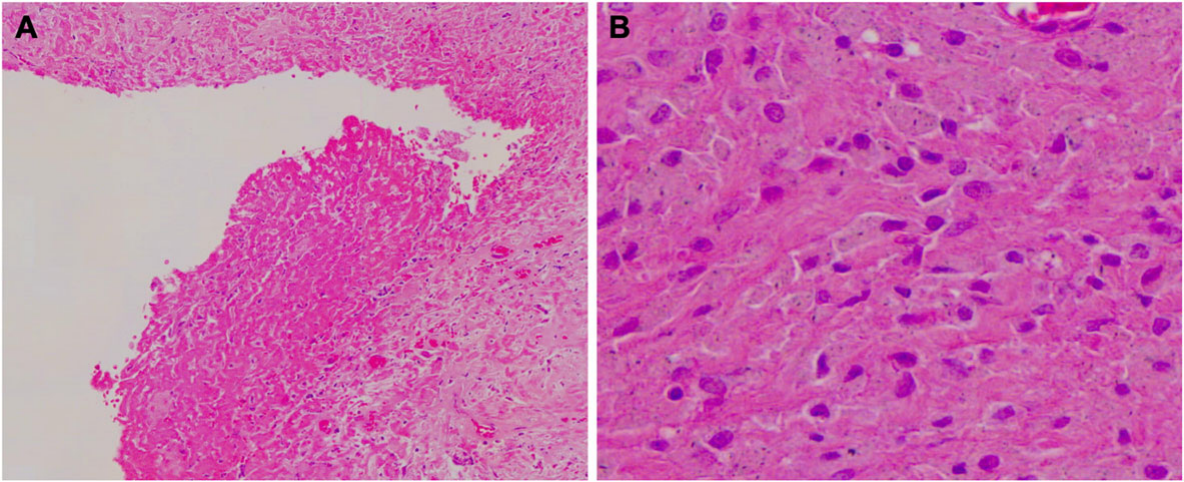
**a** The synovial lining is replaced by a fibrinous exudate (H&E, × 100). **b** Infiltrate of histiocytes with particulate material in their cytoplasm (H&E, × 400)

The joint was aspirated and blood-stained fluid was obtained which was negative on Gram staining and standard microbiological testing (culture of fluid from a normally sterile site including blood, chocolate, fastidious anaerobic and Sabouraud agar; continuous monitoring blood culture system and fastidious anaerobic enrichment broth) [4]. One month later the patient underwent explantation of the joint, with insertion of a vancomycin and meropenem impregnated articulating cement spacer. Intraoperatively, extensive damage to the ilium, ischium and pubis was noted, together with multiple turbid fluid collections in the adductor compartment and around the medial aspect of the hip. Synovial fluid alpha-defensin was negative (Synovasure™; an assay for neutrophil antimicrobial peptides (NAMP) used to support the diagnosis of PJI) [5]. Multiple intraoperative bone and tissue samples underwent histological examination and microbiological culture, and post-operatively the patient was treated empirically with meropenem and vancomycin. Antimicrobial therapy was subsequently discontinued when standard microbiological cultures were negative. While histology was not consistent with active infection, samples did show a fibrinous exudate replacing the synovial lining with abundant macrophages laden with particulate debris (Fig. 2). No granulomas were identified and no acid and alcohol fast bacilli were seen on Ziehl Neelsen staining. Following discussion at the regional bone infection multidisciplinary team meeting, residual intra-operative samples underwent broad-range bacterial 16S ribosomal deoxyribonucleic acid (rDNA) PCR, which was negative, and mycobacterial culture.

Five weeks post-operatively, tissue samples grew acid alcohol fast bacilli in liquid culture with a time to positivity of 28–31 days (BD BACTEC™ MGIT™ Automated Mycobacterial Detection System, Becton Dickson, NJ, USA). Within 5 days of the positive mycobacterial culture, the bacilli were identified by whole-genome sequencing (WGS) as *Mycobacterium bovis* (BCG lineage), and a genotypic drug susceptibility profile was determined.

**Fig. 3:**
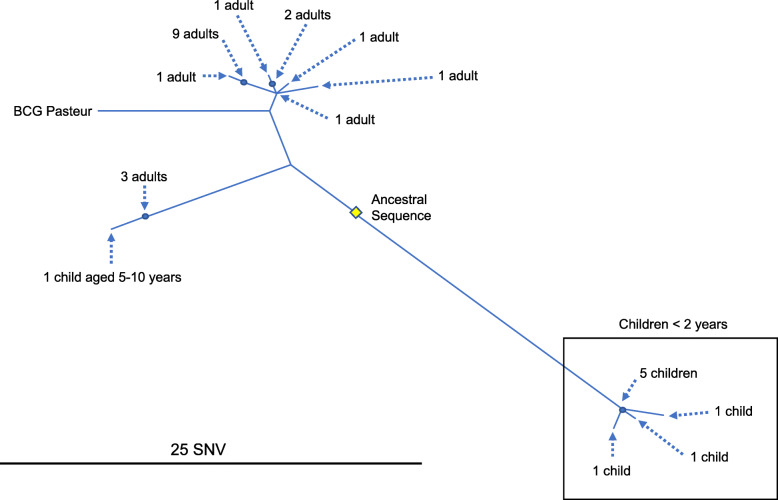
A maximal likelihood phylogeny reconstructed of 28 BCG isolates from 27 patients, isolated from clinical samples from the Midlands and North of England 2017 and 2018. A BCG Pasteur isolate is included. Identical sequences are collapsed into a single node, marked with the demographics of the cases comprising it. Our case is one of the nine adults at the top of the tree. Three distinct groups of cases are seen – children under two (right, bottom), older adults (top) and a smaller, mixed group (left)

Although we had identified BCG from intra-operative samples which was presumed to be a complication of previous intravesical therapy, we went on to further investigate the origin of the BCG by comparing our patient’s isolate with clinical BCG isolates from the Midlands and North of England (ca 15 M population) in the 2 years from January 2017 (a period during which sequencing of *M. tuberculosis* complex isolates was operational). A maximal likelihood phylogeny reconstructed using iqTree 1.6.1 with a GTR model, outgroup rooted using a lineage 1 isolate [6, 7]. We identified 28 isolates from 27 patients, which grouped into three, presumably corresponding to the BCG preparations marketed by different suppliers for vaccination or intravesical instillation (Fig. 3). The isolate received by our patient, and eight other older men who presumably also received intravesical BCG therapy, differs from the BCG strains isolated from children in the same time period (Fig. 3).

A six-month treatment regime with rifampicin, isoniazid, ethambutol and moxifloxacin was commenced (pyrazinamide was excluded due to the intrinsic resistance of *M. bovis*). The second stage revision was delayed until completion of 12 weeks of this therapy, approximately 6 months after the first stage revision (explantation), with an uncemented modular titanium femoral stem and modular titanium revision shell with metal on polyethylene articulation. The patient is presently walking without aid and without pain or clinical recurrence of PJI. 

## Discussion and conclusions

BCG is a live attenuated strain of *M. bovis* and has been used as an intravesical immunotherapy for superficial bladder cancer since 1976 [8]. Local complications of intravesical BCG therapy are well recognised and include cystitis and granulomatous prostatitis [9]. Systemic complications due to disseminated BCG infection (e.g. pneumonitis or granulomatous hepatitis) have also been described. However, reports of *M. bovis* osteoarticular infections following intravesical BCG instillation, and in particular PJI, are extremely rare.

A review of the literature suggests that only eight such cases have been reported to date, six of prosthetic hip replacements and two in prosthetic knees [9]. The time between intravesical instillation to the development of symptomatic BCG PJI ranges from 7 days to years [9–16]. It has been suggested that early infection results from haematogenous dissemination, which may be reduced by delaying BCG therapy after surgical interventions and urethral catheterisation [17]. Several hypotheses have been proposed to account for the delayed presentation of BCG PJI. BCG is often found in the urine for > 12 months after therapy so probably breaches the bladder mucosa and is transiently bloodborne, leading to seeding of the prosthesis [18, 19]. In our case the route of infection was likely haematogenous as there was no evidence of malignant spread from the bladder cancer or extension of the soft tissue abnormalities to the bladder or renal tract.

One of the common features of other cases of BCG PJI is the prolonged time between patient presentation and final diagnosis. Possible reasons include lack of clinical suspicion and appropriate diagnostic testing for mycobacteria, different patterns of inflammation relative to other bacteria, as well as the extended culture time required due to slow mycobacterial replication rate. Such delay may have significant implications for the initiation of appropriate treatment. In addition, the speciation of bacilli in mycobacterial cultures and traditional phenotypic drug sensitivity testing may increase laboratory turnaround times by several weeks.

In this case, diagnostic delay was due to low clinical suspicion and therefore mycobacterial cultures were not initially attempted. Once material was cultured in liquid cultures, and analysed by WGS, a microbiological diagnosis was rapidly achieved and appropriate surgical and medical management instituted, including targeted anti-mycobacterial chemotherapy based on genotypic drug susceptibility testing (DST) data [20]. However, WGS still relies on bacterial culture and while it offers advantages over other molecular methods of speciation, it remains relatively costly and requires complex analytical pipelines [21]. A recent advancement on the targeted amplicon sequencing used in pan-bacterial 16S rDNA PCR is shotgun metagenomic next-generation sequencing which has the potential to broaden the unbiased detection of pathogens directly from clinical samples in culture-negative PJI [22–24]. However, it is still predominantly a research technique and its widespread adoption in routine clinical diagnostics is currently limited by low yields of pathogenic DNA recovered from samples, high costs and complex data processing [25].

It should be noted that while the inclusion of synovial alpha-defensin testing, such as Synovasure™, in the diagnostic pathway for PJI is being increasingly advocated, our case demonstrates that negative NAMP assays occur in non-pyogenic infections, potentially resulting in the exclusion of PJI with implications for subsequent surgical decision-making (e.g. whether to proceed to a one or two stage revision) [5].

As this case illustrates, BCG infection after therapeutic exposure can have serious consequences, and monitoring the frequency of this complication is important. Three distinct groups of BCG isolates were identified, presumably corresponding to the products of different suppliers; WGS-based approaches to identifying the likely source may contribute to this goal, as well as unambiguously differentiating BCG from more virulent *M. bovis* isolates.

In summary, we describe the use of mycobacterial WGS for the rapid identification of *M. bovis* PJI occurring after intravesical BCG therapy. The benefits of this approach included a rapid diagnosis, definitive exclusion of infections with other *M. tuberculosis* complex members, and the timely initiation of appropriate antimicrobial therapy. Ongoing advances in sample processing, including culture-independent techniques, have the potential to reduce turnaround times further. This case also highlights the application of WGS for detecting and monitoring rare but serious complications following BCG exposure (which should be considered in PJI cases), contributing to detection and surveillance of such serious complications.

## Data Availability

All information used for this case report is available from the corresponding author.

## Abbreviations

BCG: Bacillus Calmette-Guérin
DST: Drug susceptibility testing
MRI: Magnetic resonance imaging
NAMP: Neutrophil antimicrobial peptides
PCR: Polymerase chain reaction
PJI: Prosthetic joint infection
rDNA: Ribosomal deoxyribonucleic acid
WGS: Whole-genome sequencing
